# Editorial: Assessing shift work and its health impact

**DOI:** 10.3389/fpubh.2022.1097585

**Published:** 2022-12-09

**Authors:** Yuke Tien Fong

**Affiliations:** Department of Occupational and Environmental Medicine, Singapore General Hospital, Singapore, Singapore

**Keywords:** shift work, health impact, circadian, effect, hormonal, cognition, napping

There are many work schedules that are called “shift work” (1). Shift work as explained by NIOSH, involves working outside the normal daylight hours. These workers might work in the evening, in the middle of the night, overtime or may have extralong workdays. They also might work regular days at one time or another. The work schedules might “rotate” around the clock, which involves changing work times from day to evening, or day to night. This might happen at different times of the week or at different times of the month. Police officers and firefighters, for example, often work rotating shifts. Other workers might have a “permanent” shift and only work at night or in the evenings. Waiters and waitresses, for example, might work only the evening shift. Night watchmen, on the other hand, might work only the overnight or “graveyard” shift. The IARC monogram on Night shift work (2) defined this as work during the usual sleeping hours of the general population, and included transmeridian air travel. Disruption of normal physiological circadian rhythms is the most marked effect of night shift work. In many industries, night shift work is essential for ensuring that production and activities can continue 24 h per day, 7 days per week.

Shift work is a common work pattern worldwide today but no consensus on its health impact has been made by researchers thus far. The objective of this small collection of original papers is to create a thematic approach to the subject and is aimed at summarizing the evidence from a series of original studies to highlight the impact of shift work on various aspects of the health of workers. A total of 18 manuscripts were received, of which 10 were accepted and eight rejected. Many papers have been published on the various aspects of the health impact of shift work over the years. A Medline search alone, using the key words “shift work,” “health impact” yielded 4,614 publications on the topic. Some emerging concerns have been outlined in the IARC report on the subject (2).

Shift work (3) is common in modern societies, and shift workers are predisposed to the development of numerous chronic diseases. Disruptions to the circadian systems of shift workers have been described in numerous publications and are considered important contributors to the biological dysfunction these people frequently experience. In one study (3), the authors suggest that understanding how to alter shift work and time cue schedules to enhance circadian system function is likely improve the health of shift workers.

An umbrella review (4) summarized the evidence and evaluated the validity of the associations of shift work with different health outcomes. In a search of the MEDLINE, Web of Science, and Embase databases from their inception to April 25, 2020, the authors (4) found that shift work was associated with several health outcomes with different levels of evidence. Associations for myocardial infarction and diabetes mellitus incidence were supported by highly suggestive evidence. The IARC Monographs Working Group (2) classified night shift work as “probably carcinogenic to humans” (Group 2A), on the basis of limited evidence of cancer in humans (for cancers of the breast, prostate, colon, and rectum), sufficient evidence of cancer in experimental animals, and strong mechanistic evidence in experimental animals.

An area of concern in shift work is the effect on the circadian system and the corresponding hormonal changes in the workers affected. This may have an impact on the management of occupational fatigue. In one of the papers in this series, Huang et al. explored the association between the trends of cortisol rhythm and the regularity of shift work among midwives in China. Urinary cortisol levels of participants were assayed. The results suggested that cortisol was more inhibited in midwives with irregular shift patterns and the authors opined that hospital managers may need to consider these effects while performing work scheduling for midwives. This is to minimize their occupational fatigue and in the process, this may enhance the safety of mothers and infants in their care.

While insomnia and sleepiness symptoms are common in shift workers (5), 20–30% of affected works experience more severe symptoms and meet the criteria for shift work disorder (SWD). SWD can lead to impairments in cognitive function, physical and mental health, and reduced productivity and increased risk of workplace injury. Booker et al. (5) attempted to evaluate the impact of a shift work individual management coaching program, focusing on sleep education, promoting good sleep hygiene, and providing individualized behavioral strategies to cope with shift schedules.

The effects of the use of emerging technologies on shift work was evaluated in a scoping literature review in one the studies (Bullock et al.). The findings highlight a paucity of published research on the use of mobile phone applications for sleep self-management amongst early start shift workers. Bullock et al. opined that further research is needed, on applications appropriate to this subgroup of shift workers whose unique working conditions require specific interventions and support. The appropriate timing and use of light in both the early morning and evening hours is one example of support that is specifically relevant to this group. Bullock et al. opined that while a large number of mobile phone applications that target sleep self-management already exist in the digital marketplace, few, if any, are designed specifically for use by shift workers. Is there a need, therefore, for a more evidence-based and context-specific approach to the development of mobile phone sleep applications for this group?

The mental health impact of shift work on hospital workers was explored in a cross-sectional study covering 20 hospitals in China, using a questionnaire survey (Li et al.). Li et al. opined that depression and anxiety in shift nurses may be addressed by reducing their workload, sources of stress during night shifts, and facilitating rest and relaxation.

The effects of shift work on cognition and napping was explored. Fan et al. opined that night shifts appear to have adverse cognitive outcomes that might be attenuated by daytime napping. The neurovisceral integration model suggests that resting vagally mediated heart rate variability (vmHRV) is linked with cognitive function. Fan et al. investigated the relationship between resting vmHRV and cognitive function after different nap durations in medical interns after shift work. The authors suggested that demonstrable links were found between daytime napping and improved cognitive control in relation to autonomic activity after shift work in medical interns. Li et al. suggest that autonomic activity when awake plays a crucial role in in information processing and these also affected performance testing. Autonomic activity evaluations provided more insight into understanding the differences in neurocognitive mechanisms underlying information processing after different nap durations.

In this short series, it would be impossible to cover the entire scope of the literature nor to explore the entire breadth of the dimensions on the subject. It is hoped that these highlights would stimulate more research into this engaging topic and more light will be shed on the effects of shift work on the heatlh of the worker.

## Author contributions

The author confirms being the sole contributor of this work and has approved it for publication.
